# Forest Ecosystem Service Trade-Offs/Synergies and System Function Optimization in Karst Desertification Control

**DOI:** 10.3390/plants12122376

**Published:** 2023-06-19

**Authors:** Kangning Xiong, Xuehua Deng, Shihao Zhang, Yu Zhang, Lingwei Kong

**Affiliations:** 1 School of Karst Science, Guizhou Normal University, Guiyang 550001, China; xhdeng97@163.com (X.D.); shzhang@gznu.edu.cn (S.Z.); konglw@gznu.edu.cn (L.K.); 2 State Engineering Technology Institute for Karst Desertification Control, Guiyang 550001, China; 3 Department of Resource Management, Tangshan Normal University, Tangshan 063000, China; hxcandzy@163.com

**Keywords:** functions, karst, structure–function, synergies, service, trade-offs

## Abstract

Karst desertification control forests are essential for ecosystem multi functionality, but the trade-offs/synergies are unclear for forest ecosystem services. In order to clarify the trade-offs/synergies, this study was conducted on eight forest communities in a karst desertification control area and was based on vegetation surveys and structural and functional monitoring. It analyzes water holding capacity, species diversity, soil conservation, and carbon storage characteristics and their trade-off/synergies. The results indicate the following: (1) The *Cladrastis platycarpa* + *Cotinus coggygria* community (H1) had the highest water holding capacity and species diversity with values of 252.21 t·hm^−2^ and 2.56, respectively. Soil conservation was highest in the *Zanthoxylum bungeanum* + *Glycine max* community (H6), with an index value of 1.56. Carbon storage was the greatest in the *Tectona grandis* community (H8), at 103.93 t·hm^−2^. The results of these studies have shown that there are significant differences in different types of forest community ecosystem services. (2) Water holding capacity, species diversity, soil conservation, and carbon storage, all have synergistic relationships, suggesting a trend towards synergistic enhancement between the services. (3) The species diversity of the forest ecosystems was shown to be in a trade-off with carbon storage and soil conservation, which suggests that the services are in competition with each other. To further improve the service capacity of forest ecosystems, the trade-offs between the regulation of forest community structure and function and the improvement of services should be optimized.

## 1. Introduction

As the mainstay of terrestrial ecosystems, forests have four major ecological functions: regulating, cultural, provisioning, and supporting services [1]. The regulating services of forests are climate regulation, water purification, soil conservation, and carbon storage; the cultural services are landscape, forest education, and tourism; the provisioning services are water holding, timber, charcoal, etc.; and the supporting services are species diversity, nutrient cycling, soil structure, net primary productivity, etc. [2]. Various factors regulate the services, and there are differences and overlaps in handling different services [3]. This leads to complex interrelationships between ecosystem services that manifest as synergies and trade-offs [4]. In recent decades, high–intensity economic activities and a crude forest management model, within which there is a serious lack of awareness of ecosystem services, have led to a series of problems such as reduced species diversity, soil erosion, and weakened carbon sequestration capacity [5,6]. For the purpose of enhancing forests’ ecosystem service capacity and promoting forest industry development, it is necessary to clarify the current status of different forests’ ecosystem services and clarify the trade-offs and synergistic relationships between the services.

In recent years, there has been a wealth of research on ecosystem services. There is growing interest in research related to trade-offs and synergies with ecosystem services. Qin et al. (2015) showed the trade-offs and synergies of mountain ecosystem services in Zhongguancun–Tianshui and found the highest synergies between water production and agricultural production [7]. The results of Rana et al. (2016) study showed that there are trade-offs between carbon storage, plant diversity, and some forest products in Nepalese regions and synergies between plant diversity and forest products [8]. Kearney et al. (2017) results showed synergies between supporting and provisioning services in agroforestry ecosystems, but trade-offs between provisioning and regulating services [9]. The trade−offs/synergies of forest ecosystem services are an important factor causing ecological problems [10], but research on trade-offs and synergies between forest ecosystem services is late and the research results are poor [11]. By exploring the trade-offs and synergistic relationships of services, such as water holding, species diversity, soil conservation, and carbon storage in forest ecosystems, we can reflect the current status of the services to a certain extent and adjust forest management measures according to the trade-offs, which is important for the sustainable development of forest ecosystems.

Karst desertification refers to the land degradation phenomenon caused by the disturbance and destruction of unreasonable human social and economic activities under the fragile karst environment in the subtropical zone, which is manifested by soil erosion, gradual rock exposure, land productivity degradation, and a desert–like landscape on the surface [12]. Due to the fragile ecological environment and unreasonable human activities in these areas, the problem of desertification is prominent, which greatly limits land use and economic development and directly threatens ecological security [13]. Meanwhile, human demand preferences for ecosystem services, such as excessive logging and grazing, can affect the productivity and species diversity of forest ecosystems, leading to trade-off/synergistic relationships between services and affecting their structure and function [14,15]. Therefore, in this paper, the desertification control demonstration area of Guanling–Zhenfeng Huajiang karst plateau canyon in China is taken as the study area. Based on the vegetation survey results, we divided the forest communities in the study area into eight and analyzed the water holding capability, species diversity, soil conservation, and carbon storage characteristics of the forest ecosystem. We clarified the trade-offs and synergies between forest ecosystem services and proposed functional optimization strategies. The aim was to find out the most appropriate method for the enhancement of ecological functions in the area and provide a scientific basis for sustainable regional development.

## 2. Results

### 2.1. Water Holding Characteristics of Forest Ecosystems

**Figure 1:**
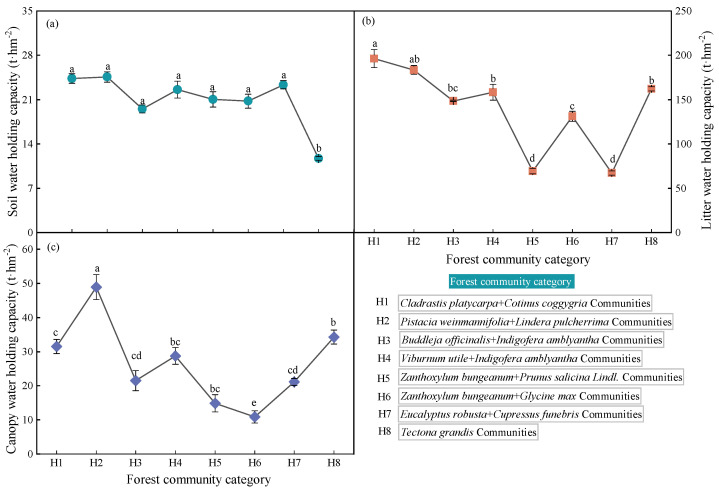
Soil, litter, and canopy water holding capacity of forest ecosystem. Note that (**a**) is soil water−holding capacity, (**b**) is litter water−holding capacity, and (**c**) is canopy water−holding capacity in the figure. The number is followed by the name of the community. Different lowercase letters indicate significant difference (*p* < 0.05).

The community with the highest soil water capacity was H2, followed by H1 and H7, with values of 24.58, 24.33, and 23.34 t·hm^−2^, respectively. The lowest community was H8, with a value of 11.69 t·hm^−2^. The community with the highest water capacity of litter was H1 with a value of 196.35 t·hm^−2^, while the community with the lowest water capacity was H7 with a value of 67.31 t·hm^−2^. The community with the highest canopy water capacity is H2, followed by H8 and H1, and the communities with the lowest canopy water capacity were H5 and H6. Overall, the order of the water conservation capacity of forest communities is ranked as H1 > H2 > H4 > H8 > H3 > H6 > H7 > H5 (Figure 1).

### 2.2. Soil Conservation Characteristics of Forest Ecosystems

The results of the improved Nemero composite index method showed that the variation in the soil composite fertility coefficients in the karst plateau canyon forest ecosystem ranged from 0.904 to 1.562, and all the soil fertility belonged to the average grade. From the fertility coefficients of each soil attribute, TP, TN, and SOM were lower in the H8 communities and TK was lower in the H1 and H2 communities, implying that the H8 communities were vulnerable to TP, TN, and SOM, whereas the H1 and H2 communities were vulnerable to TK. In addition, the overall low AP coefficients of all forest communities showed the lack of AP nutrients during plant growth, which may need to be supplemented with emphasis. In terms of the integrated soil fertility coefficient, the best fertility was in the H5 community with an index value of 1.562, and the worst fertility was in the H8 community with an index value of 0.905. The reason was that the H8 community had rocky outcrops, a thin soil layer, and severe nutrient loss, resulting in poor soil fertility (Table 1).

### 2.3. Forests Ecosystem Species Diversity Characteristics

**Figure 2:**
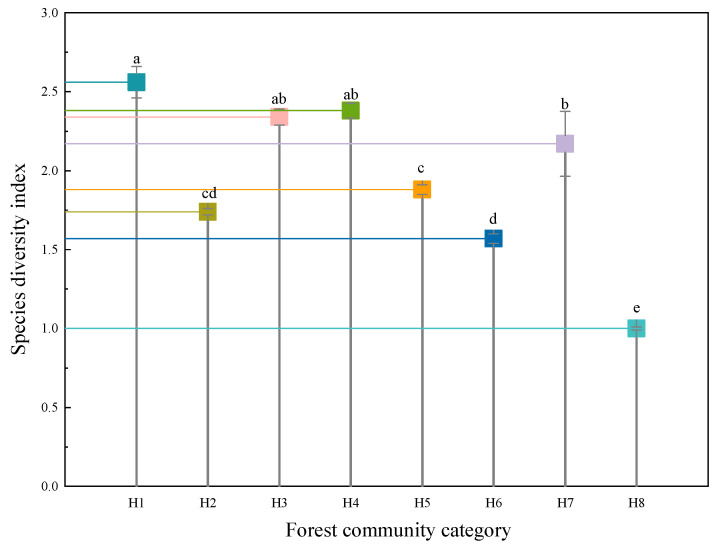
Species diversity index of forest ecosystem. Note: different lowercase letters indicate significant difference (*p* < 0.05).

The results based on the species diversity index method showed that the H1 community had the highest species diversity index (2.56) and was significantly different from the other forest communities, which was reflected its superior ecological recovery and its species diversity, which was the richest. This was followed by communities H4 (2.38) and H3 (2.34), which consisted of shrubs and herbs with high vegetation cover and species richness. The community with the lowest species diversity index was H8 (1.00), which was a teak plantation, with tall trees with dense canopies, significant shading, and a bad environment in the forest, making it difficult for species to grow (Figure 2).

### 2.4. Forest Ecosystem Carbon Storage Characteristics

**Figure 3:**
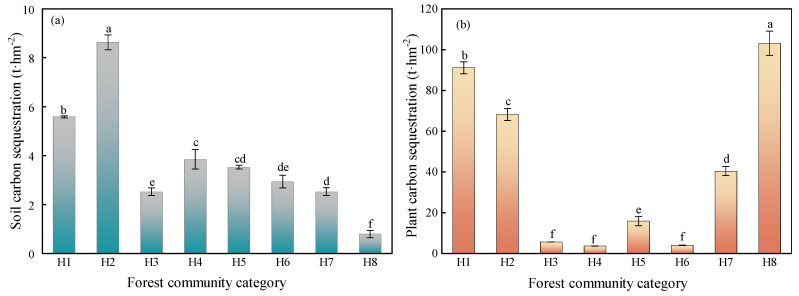
Soil and plant carbon sequestration of forest ecosystems. Note that (**a**) is soil carbon sequestration and (**b**) is plant carbon sequestration. Different lowercase letters indicate significant difference (*p* < 0.05).

The soil carbon stock and plant carbon stock of each forest community had significant differences (Figure 3). The distribution of their soil carbon stocks ranged from 0.79 to 8.63 t·hm^−2^, and the distribution of the plant carbon stocks ranged from 3.7 to 103.13 t·hm^−2^. Among them, the H2 and H1 communities had the highest soil carbon stocks, which were 10.83 and 7.01 times higher than those of the H8 community. The H8 community had higher plant carbon stocks, followed by the H1 and H2 communities, and the H4 and H6 communities had lower stocks. Collectively, the carbon stock size of the forest ecosystem was ranked as the H8 > H1 > H2 > H7 > H5 > H3 > H4 > H6 communities.

### 2.5. Trade-Offs and Synergies among Forest Ecosystem Services

The correlation analysis shows that there is a synergistic relationship between water holding capacity and species diversity, carbon storage, and soil conservation in forest ecosystems. There is also a synergistic relationship between carbon storage and soil conservation, reflecting a synergistic trend among species diversity, carbon storage, and soil conservation in forest ecosystems. In addition, there is a trade−off between forest ecosystem species diversity, carbon storage, and soil conservation, indicating that these services are in a competitive state and have a significant impact on the supply capacity of the services (Table 2).

## 3. Materials and Methods

### 3.1. Study Sites

**Figure 4:**
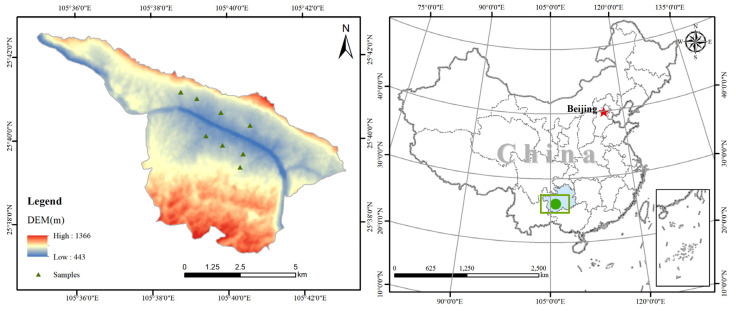
Location of the study area. Note: the red star is the capital of China, and the green dot is the location of the study area.

The study area is located in Guanling County, Anshun City, Guizhou Province, on the banks of the Huajiang Gorge in Huajiang Town (105°41′30.09″ E, 35°39′49.64″ N). The habitat is a dry−heat river valley desertification area. The valley is deep; the highest elevation is 1473 m and the lowest is 550 m, with a relative elevation difference of 943 m. It is a river valley climate, with warm winters and springs, hot and humid summers and autumns, and an annual average temperature of about 18.4 °C, with abundant heat resources. The average annual rainfall is 1100 mm and has an uneven seasonal distribution. Rainfall is mostly concentrated in the summer, with severe drought in winter and spring caused by volcanic activity. The type of rock desertification in the study area is mainly moderate intensity, with a high rate of bedrock exposure, broken surface, poor soil, mainly limestone soil, low vegetation cover, and predominantly natural secondary forests (Figure 4).

### 3.2. Treatment Setting

During the peak plant growth period in July–August 2022, eight forest communities in the study area were selected for their relative goodness within the study area, based on the state of forest recovery. These were the *Cladrastis platycarpa* + *Cotinus coggygria* community (H1), *Pistacia weinmannifolia* + *Lindera pulcherrima* community (H2), *Buddleja officinalis* + *Indigofera amblyantha* community (H3), *Viburnum utile* + *Indigofera amblyantha* community (H4), *Zanthoxylum bungeanum* + *Prunus salicina* Lindl. community (H5), *Zanthoxylum bungeanum* + *Glycine max* community (H6), *Eucalyptus robusta* + *Cupressus funebris* community (H7), and *Tectona grandis* community (H8). In each community, 20 m × 20 m plots were delineated and recorded; information was recorded on their slope, slope directions, species name, diameter at breast height, plant height, and crown width (Table 3).

### 3.3. Sample Collection and Index Determination

#### 3.3.1. Sample Collection

Based on a vegetation survey, the dominant species in the sample plots were selected, and 3–10 healthy, mature fresh leaves were marked and placed in plastic bags, which were then taken back to the laboratory to determine their water holding capacity. A small sample of 1 m × 1 m litter was taken from each sample plot and placed in a nylon mesh bag, marked, and taken back to the laboratory to determine its water holding capacity. Three samples of 0–20 cm soils were collected with a ring knife (100 cm^3^), marked with the sample plot number and the weight of the ring knife, and brought to the laboratory to determine the physical properties of the soil (water holding capacity and soil capacity). In addition, 5–7 sampling points were selected in an “S” pattern from the 0–20 cm soil layer after the removal of litter and impurities to obtain a mixed sample of approximately 500 g for the determination of soil chemical characteristics organic matter, total phosphorus, fast−acting phosphorus, total nitrogen, total potassium, and pH.

#### 3.3.2. Index Determination

Canopy water holding capacity: The leaves in the plastic bag were weighed and placed in a nylon net, and then, the leaves in the nylon net were all fully soaked in water for 24 h before being removed to drain out the excess water, which was said to define the maximum water holding capacity; Withered litter water holding capacity: The withered litter collected in the field was put into non−absorbent nylon mesh bags and marked. The mesh bags were completely immersed in water and taken out after 24 h. They were then weighed, and the time at which the mesh bags were no longer dripping was recorded. Finally, they were placed in an oven with the temperature adjusted to 75 °C and baked for 48 h. Then, they were taken out and weighed.

Soil physical properties: The ring knife soil was weighed for its fresh weight and placed on a tray. Water (not exceeding the ring knife cover) was poured into the tray, and the water was held for 24 h and weighed and recorded, respectively. After that, gravel and filter paper were placed in the tray, and the ring knife was placed on the tray after holding water and weighed after infiltration for 8 h. When the above steps were completed, we placed the ring knife soil in the soil oven with the temperature set to 105 °C and the time set to 24 h to dry, and then, it was weighed and recorded.

Soil chemical properties: The chemical soils brought back to the laboratory from the field were stripped of impurities, ground after natural drying, and passed through 2 and 0.15 mm sieves, respectively. The sieved soil samples were kept in sealed bags, marked, and kept in a cool place. Soil organic matter (SOM) was measured using the potassium dichromate bulk density method and the external heating method. Total phosphorus (TP) was determined by perchloric acid sulfuric acid−digestion molybdenum antimony anti−colorimetry ultraviolet spectrophotometry. Available phosphorus (AP) was determined by hydrochloric acid−ammonia fluoride extraction–molybdenum antimony resistance colorimetry. Total nitrogen (TN) was measured by the semi−micro Kjeldahl method. Total potassium (TK) was measured using the sodium hydroxide fusion flame photometer method [16]. A soil pH meter (pH268, Sigma Instruments Co., Ltd., Beijing, China) was used to detect soil acidity and alkalinity.

### 3.4. Research Methods

(1) Water conservation:

*WC* = *WL* + *R_max_* + *SH* (1)

where *WC* is water holding capacity, *WL* is withered litter water holding capacity, *R_max_* is canopy water holding capacity, and *SH* is soil water holding capacity.

  ① Withered litter water holding capacity computation:

(2) WL=(W1−W2)/W2

where *WL* is withered litter water holding capacity, *W*1 is the maximum water holding capacity of the litter after 24 h of soaking in water, and *W*2 is the weight of the litter after 24 h of drying.

  ② Canopy water holding capacity computation:

(3) $$R_{\max}=\frac{\left(G-G0\right)}{G0}\times 100\%$$

where *R_max_* is the effective water holding rate, *G*0 is the fresh weight of the leaf, and *G* is the weight of the maximum water holding state of the leaf.

  ③ Soil water holding capacity computing:

(4) $$SH=\frac{Y_{i}-D_{i}}{D_{i}}\times 100\%$$

where *SH* is the field water holding capacity, *Y_i_* is the wet soil weight, and *D_i_* is the dry soil weight.

(2) Soil Conservation:

The modified Nemero composite index method was used for comprehensive evaluation [17], and seven indicators, pH, SOM, TN, TP, TK, AP, and soil capacity (*ρ*b), were selected as reference terms to reflect the fertility status of the soil comprehensively. First, the seven parameters were standardized to eliminate the difference in magnitudes among the parameters. The calculation formula was as follows:

(5) $$\left\{ \begin{array}{l} P_{i}=C_{i}/X_{a}\;\;\;\;\;\left(P_{i}\leq 1\right)\;\;\;\;C_{i}\leq X_{a}\\ P_{i}=1+\frac{C_{i}-X_{a}}{X_{b}-X_{a}}\;\;\;\left(1<P_{i}\leq 1\right)\;\;\;X_{a}<C_{i}\leq X_{b}\\ P_{i}=1+\frac{C_{i}-X_{a}}{X_{b}-X_{a}}\;\;\;\left(2<P_{i}\leq 3\right)\;\;\;X_{b}<C_{i}\leq X_{c}\\ P_{i}=3\;\;\;\;\;\;\;\;\left(C_{i}>X_{c}\right) \end{array}\right.$$

where *P_i_* is the soil fraction fertility coefficient; *C_i_* is the attribute determination value; and *X_a_*, *X_b_*, and *X_c_* are the graded standard values of each soil attribute (Table 4) [18].

The soil capacity was in the range of 1.14–1.26 g/cm^3^, which is better for the emergence of seedlings and normal root growth because a range greater or smaller than this would have a smaller fertility coefficient after standardization [18]. Therefore, the standardization of the capacitance was carried out with special treatment. The standardization method was as follows:

(6) $$\left\{ \begin{array}{ccc} F_{i}=\frac{1.45}{C_{i}} & \left(F_{i}\leq 1\right) & \left(C_{i}\geq 1.45\;g/cm^{3}\right)\\ F_{i}=1+\frac{C_{i}-1.45}{1.35-1.45} & \left(1<F_{i}\leq 2\right) & \left(1.35\;g/cm^{3}\leq C_{i}<\;g/cm^{3}\right)\\ F_{i}=2+\frac{C_{i}-1.35}{1.25-1.35} & \left(2<F_{i}\leq 3\right) & \left(1.25\;g/cm^{3}\leq C_{i}<1.35\;g/cm^{3}\right)\\ F_{i}=3 & & \left(1.14\;g/cm^{3}\leq C_{i}<1.25\;g/cm^{3}\right) \end{array}\right.$$

After standardizing the indicators of this study using the above standardization method, the fertility coefficients obtained for each sub−fertility were used for further comparison and calculation. Finally, the modified Nemero formula was used for the calculation of integrated fertility.

(7) $$F=\frac{\sqrt{F_{i}^{2}+{F_{i\min}}^{2}}}{2}*\left(\frac{n-1}{n}\right)$$

where *F* is the integrated fertility coefficient of soil; *F_i_* is the average value of the fertility coefficient of each attribute of soil; *F_imin_* is the minimum value of the fertility coefficient of each attribute of soil; and *n* is the number of soil indicators evaluated.

Where the *F_i_* minimum replaces the *F_i_* maximum in the previous Nemero equation, a correction term (*n* − 1)/*n* is added to the original equation. On the one hand, this highlights the effect of the worst of the soil properties on fertility. On the other hand, with the addition of the correction term, the credibility of its evaluation results increases, and it is more scientific [19]. Soil fertility coefficients were obtained according to the Nemero formula, and the soil was graded for fertility (Table 5).

(3) Species diversity:

The overall community species diversity index conversion formula is [20]

(8) $$W_{i}=\left(\frac{C_{i}}{C}+\frac{H_{i}}{H}\right)/2$$

where *C* is the total community cover, the tree layer is *i* = 1, the shrub layer is *i* = 2, and the herb layer is *i* = 3; *H* is the height of each growth type of the community; *W_i_* is the diversity of the *i*th growth type of the community; *C_i_* is the cover of the ith growth type; and *H_i_* is the height of the ith growth type.

(4) Carbon storage:

*C_total_* = *C_vagetation_* + *C_soil_* (9)

where *C_total_* is the forest ecosystem carbon stock; *C_vagetation_* represents plant carbon stock; and *C_soil_* represents soil carbon stock.

  ① Carbon sequestration by plant computing:

Determine the coefficient of the conversion of biomass to organic carbon from the photosynthesis equation.

CO_2_ (264g) + H_2_O → C_6_H_12_O_6_ (180g) + O_2_ (192g) → Polysaccharide (162g) (10)

The above equation shows that for every 1 g of dry matter produced, 1.63 g of CO_2_ is required, of which, C accounts for 27.27%.

  ② Soil carbon sequestration computation:

(11) $${\;C}_{\mathrm{soil}}=A_{i}\times S_{c}$$

where: *C_soil_* is the amount of carbon sequestration in the soil of the assessed forest; *A_i_* is the area of forest type *i*, 0.04 (hm^2^); and *S_c_* is the carbon storage of the measured forest soil per unit area (t·hm^−2^) [21].

### 3.5. Data Analysis

Using Microsoft Excel 2016 software to preliminarily organize the data, the significant differences among water conservation, species diversity, and carbon storage in forest ecosystems were tested using SPSS22.0 one−way ANOVA, and the results were expressed as mean ± standard deviation. Pearson correlation analysis was used to examine the relationship between water conservation, species diversity, carbon storage, and soil conservation in forest ecosystems in karst rocky desertification areas. For mapping, we used Origin (2018) software.

## 4. Discussion

### 4.1. Forest Ecosystem Services for Karst Desertification Control

Forests are the main body of terrestrial ecosystems, providing different types of ecosystem services for the maintenance of life on Earth such as water holding, species diversity, and soil conservation [22]. In this study, the litter water holding capacity of the H1 community in the karst desertification area is higher than that of other communities, while the soil and canopy water holding capacities are lower than those of other communities; however, the water conservation capacity is the strongest. The reason is that the H1 community belongs to a secondary forest that has been well restored under closed mountain cultivation. The three−dimensional structure of trees, shrubs, and grasses is significant. The denser forest canopy has a strong protective effect on forest water, a humid environment under the forest, a high degree of litter decomposition, and a strong water conservation ability [23]. Soil fertility is an important basis for measuring soil health, which can comprehensively reflect soil physical and chemical properties, maintain the long−term productivity of plants, and have a certain stabilizing effect on nutrient cycling in the ecosystem [24]. Our results showed that the H5 community has the highest comprehensive soil fertility index, indicating that its soil conservation ability is higher than other communities, which may be caused by artificial fertilization. The H5 community is a pioneer for vegetation restoration in karst plateau canyons [25]. Growers have improved soil fertility and promote the growth of the H5 population by regularly applying organic and chemical fertilizers to the plants [14].

Tall trees have higher carbon storage [26]. This is consistent with our results, as the H8 community had the highest carbon storage, but its species diversity and soil conservation ability were both poor. The reason may be that the H8 community is composed of tall teak plantations, with a rapid growth rate, robust branches, and high carbon storage. However, the structure of the teak community is single, with a large crown diameter, and poor light transmittance within the forest [27]. There is a fiercely competitive relationship between the lower vegetation and the upper trees in the forest [28]. In addition, improper tending measures (such as excessive use of herbicides, etc.) have led to a decline in the stability of the plantation ecosystem and a decrease in species diversity [27]. In addition, H8 communities grow in areas of intense rocky desert throughout, with shallow soil layers and flowing water. Soil nutrients should not be retained [29], and the soil conservation capacity is significantly lower than other forest communities. To sum up, different communities have different ecosystem service capabilities, which are mainly caused by differences in community structure, resource utilization methods, and adaptation strategies to habitats. 

### 4.2. Trade−Offs/Synergies of Forest Ecosystem Services for Karst Desertification Control

Changes in the ecosystem structure will cause corresponding changes in the ecosystem services and functions, thereby affecting the trade−off and synergy between the ecosystem services or functions [30]. In this study, we found a synergistic relationship between water conservation and species diversity, carbon storage, and soil conservation in forest ecosystems, as well as a synergistic relationship between carbon storage and soil conservation. It indicates that there is a positive synergy between water conservation and species diversity, carbon storage, and soil conservation in forest ecosystems, which can significantly enhance the service supply capacity of forest ecosystems, consistent with Valentine’s research results [31]. The reason is that the demonstration area for the comprehensive control of rocky desertification in the canyons of the karst plateau has been under control for a long time. After implementing policies such as the project of returning farmland to forests and grasslands, the project of the comprehensive control of rocky desertification, and the closing of mountains for forest cultivation, the demonstration area has gradually changed from a “rocky desert mountain” to a “green mountain”, and the stability of the forest ecosystem has increased. In addition, after the implementation of the policy of stopping using mountains for forest cultivation, the degree of human interference has decreased, the soil erosion resistance ability of the forest ecosystem has increased, and the microbial activity in the soil is high [32]. Trees, shrubs, and grasses grow rapidly, significantly increasing vegetation coverage, thereby enhancing the species diversity, carbon fixation capacity, and canopy interception capacity of forest communities [33]. The above functions are important components for identifying the water conservation capacity of forests, so their synergistic relationship is significant.

Zhou et al. (2022) believe that there is no significant relationship between species richness and above−ground carbon storage, but there is a strong negative correlation between plant diversity (measured by species diversity index) and above−ground carbon storage [29]. Qin et al. (2021) also believe that there is a trade−off relationship between forest carbon and plant diversity [34]. This study is consistent with the above results, which may be related to tree density and size. Large trees play a more important role in forest carbon storage in karst desertification areas. The selective removal of large trees for forest thinning may reduce carbon without negatively affecting species diversity [8]. In addition, studies have shown that ecosystem functions (such as carbon storage) are not only determined by the number of species but more likely by the characteristics of existing species; that is, the species composition or functional characteristics of specific species are more predictive of ecosystem functions than species richness [35]. Specific species in karst desertification areas also exhibit similar characteristics [36], such as *Cladrastis platycarpa*, *Cunninghamia lanceolata*, *Tectona grandi*, *Cotinus coggygri*, etc. After adapting to the special habitats in karst rocky desertification areas, their growth rate is faster, and their carbon fixation ability is superior to other species [20]. However, the mechanism of how specific functional characteristics of species affect ecosystem service capacity needs to be further studied.

### 4.3. Optimization Strategy of Forest Ecosystem Service Function for Karst Desertification Control

**Figure 5:**
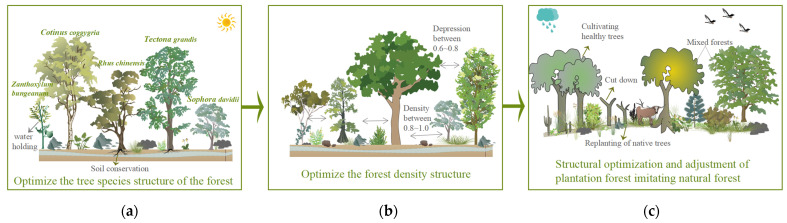
Forest ecosystem functions optimization strategy. Note: (**a**) is optimize the structure of tree species in the stand, (**b**) is optimize the structure of stand density, and (**c**) is the structure of plantation forests imitates natural forests to optimize the regulation.

The forest stand structure in karstic stone desertification areas is homogeneous, species diversity is low, and ecosystem functions are gradually degraded, which affects the sustainable and stable development of forest ecosystems [37]. To reduce the competitive dynamics among plants, improve the structure and function of the ecosystem, and enhance the service capacity of the forest ecosystem, the forest ecosystem service function can be optimized from the following aspects (Figure 5).

**Figure 6:**
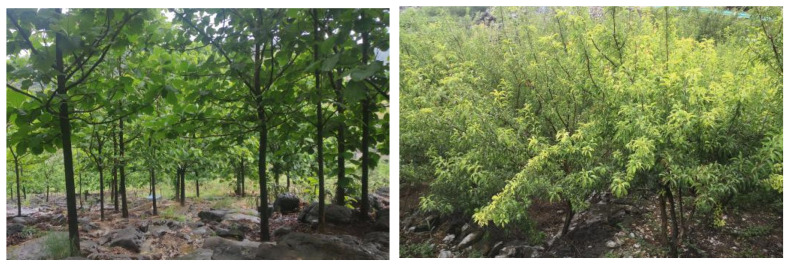
Optimize the forest density structure.

**Figure 7:**
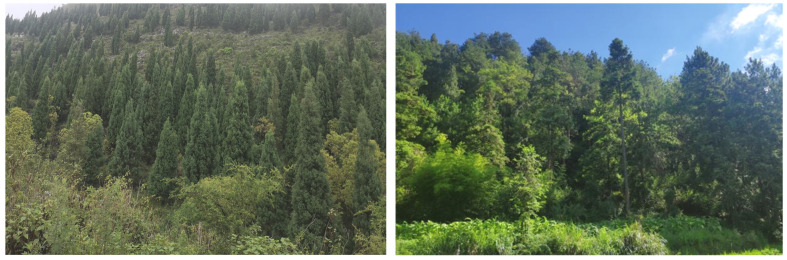
Structural optimization and adjustment of plantation forest imitating natural forest.

1. Optimize the structure of tree species in the stand: when selecting tree species, it is recommended to choose species with strong soil−holding and water holding capacities, whose trees are mainly *Cladrastis platycarpa*, *Cotinus coggygria*, *Lindera pulcherrima*, *Tectona grandis*, *Cupressus*, and *Rhus chinensis*. In addition, trees with waxy leaves such as *Eriobotrya japonica*, *Yulania denudata*, and other plants with high water holding capacity can be introduced [38]; shrubs are mainly selected from *Zanthoxylum bungeanum*, *Sophora davidii*, *Pistacia weinmannifoli*, *Prunus salicina* Lindl., *Indigofera amblyantha*, and *Celtis sinensis*, among other species [39].
2. Optimize the structure of stand density: for natural forests with high stand density, the stand density should be controlled between 0.8 and 1.0 by adopting inter−felling and branching, and the degree of depression should be controlled between 0.6 and 0.8. Enlarging the distance between stands reduces the number of plants per unit area so that plants are given sufficient light conditions and growing space [40] (Figure 6).
3. The structure of plantation forests imitates natural forests to optimize the regulation: Most forests in the karst stone desertification control area are artificial forests, and the spatial structure of forest stands should be adjusted according to the original forest or the spatial structure of forest stands close to the original forest [41]. The tree species composition of the stand is determined according to the stand conditions and the climate zone in which the stand is located, and the tree species composition of the stand is adjusted by the method of “cutting small and leaving big, cutting dense and leaving thin”, which preserves trees with a straight and complete stem shape and large diameter at breast height and harvests small trees with poor growth and a poor stem shape. We also adjusted the angular scale of retained trees to increase the proportion of structural units with values of 0.75 and 1 and replanted other native species in the forest gaps by harvesting some of the nearest neighboring trees. It is important to plant other suitable native species in the canopy and gaps of planted forests and promote forest regeneration to change the status quo of single species in forest stands and gradually form a mixed multi−species state to reduce the emergence of trade−off relationships (Figure 7).

### 4.4. Shortcomings and Progress

This article is limited to research on water conservation, soil conservation, carbon storage, and species diversity. Water purification, climate regulation, forest protection, ecological cultural tourism functions, etc., have not been mentioned in the article. In order to fully understand the current situation of forest ecosystem services for karst desertification control and formulate better structural and functional optimization strategies, more research on forest ecosystem services for karst desertification control should be carried out in the future. In addition, this study only analyzes the trade−offs/synergies of forest ecosystem services for karst desertification control, but the internal mechanisms and impact mechanisms of the interactions of various services have not been thoroughly analyzed. In the future, the response of trade−offs and synergistic relationships between different ecosystem services to each de−driving mechanism should be explored more intensively. In particular, we should focus on the intrinsic ecological factors and quantify the influence of different stand types, structural characteristics, and growth and development processes on the capacity of forests to provide services and the interrelationship of each service.

## 5. Conclusions

In this paper, we studied the ecosystem service capacity and trade−off synergistic characteristics of eight forest communities in karst stone desertification areas with the following conclusions:

(1) The *Cladrastis platycarpa* + *Cotinus coggygria* community (H1) and the *Pistacia weinmannifolia* + *Lindera pulcherrima* community (H2) have the strongest water holding capacity, followed by the *Viburnum utile* + *Indigofera amblyantha* community (H4) and the *Tectona grandis* community (H8). The *Zanthoxylum bungeanum* + *Prunus salicina* Lindl. community (H5) and the *Eucalyptus robusta* + *Cupressus funebris* community (H7) have the worst water holding capacity.
(2) The variation in soil fertility coefficients in forest ecosystems ranged from 0.904 to 1.562, and the soil fertility was of average grade. The best soil fertility index was for the *Zanthoxylum bungeanum* + *Prunus salicina* Lindl. (H5) communities (1.562), and the worst was for the *Tectona grandis* community (H8) (0.905).
(3) The variation in the species diversity index of the forest ecosystem ranged from 1 to 2.56, among which, the *Cladrastis platycarpa*+ *Cotinus coggygria* community (H1) had the highest species diversity index (2.56), followed by the *Viburnum utile* + *Indigofera amblyantha* community (H4) (2.38) and the *Buddleja officinalis* + *Indigofera amblyantha* community (H3) (2.34) communities, and the lowest species diversity index was in *Tectona grandis* community (H8) (1.00).
(4) The distribution of soil carbon stock in forest ecosystems ranged from 0.79 to 8.63 t·hm^−2^, and the distribution of plant carbon stock ranged from 3.7 to 103.13 t·hm^−2^. The overall forest ecosystem carbon stock size was ranked as the *Tectona grandis* community (H8) > *Cladrastis platycarpa*+ *Cotinus coggygria* community (H1) > *Pistacia weinmannifolia* + *Lindera pulcherrima* community (H2) > *Eucalyptus robusta* + *Cupressus funebris* community (H7) > *Zanthoxylum bungeanum* + *Prunus salicina* Lindl. community (H5) > *Buddleja officinalis* + *Indigofera amblyantha* community (H3) > *Viburnum utile* + *Indigofera amblyantha* community (H4) > *Zanthoxylum bungeanum* + *Glycine max* community (H6).
(5) There is a synergistic relationship between water conservation, species diversity, carbon storage, and soil conservation in forest ecosystems; carbon storage and soil conservation also show a synergistic relationship. There is a trade-off relationship between species diversity, carbon storage, and soil conservation.
(6) Karst stone desertification control forests can optimize ecosystem service relationships and enhance forest ecosystem service capacity by regulating tree species structure, density structure, and plantation imitating natural forest structures.

## Figures and Tables

**Table 1:** Comprehensive soil fertility of the karst plateau canyon forest.

Sample Number	Soil Fraction Fertility Factor							Integrated Fertility Factor *F*	Fertility Grade
	TP/ (g·kg^−1^)	TK/ (g·kg^−1^)	TN/ (g·kg^−1^)	SOM/ (g·kg^−1^)	AP/ (mg·kg^−1^)	*ρ*b/ (g·cm^−3^)	pH		
H1	2.82	0.22	3.00	3.00	0.28	1.81	2.09	1.152	III
H2	3.00	0.22	3.00	3.00	0.17	3.00	3.00	1.336	III
H3	3.00	0.82	3.00	3.00	0.08	0.99	3.00	1.204	III
H4	3.00	4.02	3.00	3.00	0.10	1.88	3.00	1.561	III
H5	3.00	0.99	3.00	3.00	0.59	0.98	6.00	1.562	III
H6	3.00	1.03	3.00	3.00	0.39	0.82	3.00	1.254	III
H7	2.02	0.81	3.00	3.00	0.25	0.98	3.00	1.142	III
H8	0.72	1.07	1.53	1.70	0.34	1.87	2.95	0.905	III

**Table 2:** Trade−offs and synergies among forest ecosystem services.

Service Category	Water Holding Capacity	Species Diversity	Carbon Storage	Soil Conservation
water holding capacity	1			
Species diversity	0.24	1		
Carbon storage	0.513 *	−0.298	1	
Soil conservation	0.261	−0.05	0.209	1

Note: * indicates significant difference (*p* < 0.05).

**Table 3:** Basic characteristics of plots.

Site	Slope/°	Aspect/°	Mean Crown/m	Mean Tree Height/m
H1	20	Southeast 145°	3.2 × 3.1	7.45
H2	26	Southeast 101°	3.1 × 2.9	4.63
H3	30	East 102°	0.8 × 1.1	1.04
H4	28	Northwest 280°	1.20 × 1.1	1.47
H5	10	Southwest 232°	1.35 × 1.33	1.43
H6	18	North 22°	1.12 × 0.92	3.45
H7	30	Southwest 220°	3.0 × 2.8	8.03
H8	19	Southeast 149°	3.3 × 2.9	8.35

**Table 4:** Grading standard values of soil properties [18].

Soil Properties	*X_a_*	*X_b_*	*X_c_*
TN (g·kg^−1^)	0.75	1.50	2.00
TP (g·kg^−1^)	0.40	0.60	1.00
TK (g·kg^−1^)	5.00	20.00	25.00
SOM (g·kg^−1^)	10.00	20.00	30.00
AP (mg·kg^−1^)	5.00	10.00	20.00
*ρ*b	1.45	1.35	1.25
pH (≤7.0)	4.5	5.5	6.5
pH (>7.0)	9.0	8.0	7.0

Note: *X_a_*, *X_b_*, and *X_c_* are the grading standards for attributes.

**Table 5:** Comprehensive soil fertility grades.

Soil Fertility Grade	Grade I (Very Fertile)	Grade II (Fertile)	Grade III (General)	Grade IV (Barren)
Fertility coefficient range	≥2.70	2.70~1.80	1.80~0.90	<0.90

## Data Availability

The data presented in this study are available in the article.
